# The Proteomes of Oral Cells Change during Co-Cultivation with *Aggregatibacter actinomycetemcomitans* and *Eikenella corrodens*

**DOI:** 10.3390/biomedicines11030700

**Published:** 2023-02-24

**Authors:** Boris Schminke, Philipp Kauffmann, Phillipp Brockmeyer, Nicolai Miosge, Christof Lenz, Andrea Schubert

**Affiliations:** 1 Department of Oral and Maxillofacial Surgery, University Medical Center, 37075 Goettingen, Germany; 2 Department of Prosthodontics, University Medical Center, 37075 Goettingen, Germany; 3 Department of Clinical Chemistry, University Medical Center, 37075 Goettingen, Germany; 4 Bioanalytical Mass Spectrometry Group, Max Planck Institute for Multidisciplinary Sciences, 37077 Goettingen, Germany

**Keywords:** proteome, gingival keratinocyte, osteoblastic lineage cell, PDL fibroblast, cementoblast, bacteria

## Abstract

Background: Changes in the proteome of oral cells during periodontitis have rarely been investigated. This lack of information is partially attributed to the lack of human cell lines derived from the oral cavity for in vitro research. The objective of the present study was to create cell lines from relevant oral tissues and compare protein expression in cells cultured alone and in cells co-cultivated with periodontitis-associated bacterial strains. Methods: We established human cell lines of gingival keratinocytes, osteoblastic lineage cells from the alveolar bone, periodontal ligament fibroblasts, and cementum cells. Using state-of-the-art label-free mass spectrometry, we investigated changes in the proteomes of these cells after co-cultivation with *Aggregatibacter actinomycetemcomitans* and *Eikenella corrodens* for 48 h. Results: Gingival keratinocytes, representing ectodermal cells, exhibited decreased expression of specific keratins, basement membrane components, and cell-cell contact proteins after cultivation with the bacterial strains. Mesodermal lineage cells generally exhibited similar proteomes after co-cultivation with bacteria; in particular, collagens and integrins were expressed at higher levels. Conclusions: The results of the present study will help us elucidate the cellular mechanisms of periodontitis. Although co-cultivation with two periodontitis-associated bacterial strains significantly altered the proteomes of oral cells, future research is needed to examine the effects of complex biofilms mimicking in vivo conditions.

## 1. Introduction

The periodontium is formed by the gingiva, cementum, periodontal ligament (PDL), and alveolar bone [1]. These soft and hard tissues are essential for tooth attachment and proprioception during mastication [2]. Moreover, periodontal tissues constitute a barrier against the oral microbiome, the penetration of which can lead to inflammation and periodontal disease [3]. Alveolar bone is composed of a few cell types, namely, osteocytes, which exhibit direct cell–cell contact, osteoblasts, and osteoclasts [4]. These cells are embedded in a vascularized, calcified extracellular matrix (ECM). Typical proteins of the alveolar bone ECM are collagen types I, III, V, and VI [5]. The composition of the cementum is similar to alveolar bone. Cementum surrounds the tooth root and is connected to the PDL. Cementoblasts and cementocytes also produce ECM proteins, mainly collagen types I and III [6]. The PDL mediates masticatory forces between alveolar bone and tooth; its ECM is produced by fibroblasts and is mainly composed of collagen types I [7], III, V, VI, and XII [2], oxytalan fibers [8], fibulins, and matrilins [9]. The gingiva is an epithelial tissue composed of gingival keratinocytes with a typical keratin pattern [10] and a small amount of ECM that mainly consists of glycosaminoglycans [11]. Although the structural composition of periodontal tissues is well described in the literature, little is known about the proteome and the molecular mechanisms of periodontal cells in health and disease. Periodontitis is defined as the chronic inflammatory disease of periodontal tissues [12] and is currently the most common disease in patients over 30 years of age [13]. The etiology of periodontitis is multifactorial; it includes a genetic predisposition, smoking, diabetes, an impaired host response, stress, and insufficient oral hygiene [14,15]. Periodontitis is the primary reason for tooth loss and is characterized by the irreversible destruction of the periodontium [3]. Periodontal destruction is initiated by microorganisms in the biofilm and the corresponding immune response, particularly infiltrating neutrophils [16]. This cascade leads to the synthesis of inflammatory mediators that collectively contribute to tissue destruction and bone resorption, including cytokines, chemokines, arachidonic acid metabolites, and proteolytic enzymes [17]. Two major pathogenic microorganisms associated with periodontitis are *Aggregatibacter actinomycetemcomitans* and *Eikenella corrodens* [14,18,19]. Both produce virulence factors that promote the rapid dismantling of the ECM of the periodontium [20,21].

Although periodontitis is a widespread disease, its molecular mechanisms in human oral cells are not completely understood. However, it is known that periodontitis as a host-mediated disease can be influenced by autologous proteins such as melatonin, which is able to reduce pocket depth and clinical attachment loss [22]. The importance of host-mediated disease is further emphasized when peri-implant marginal bone loss is evaluated. Here, in a similar bacterial setting, increased levels of MMP8 as a bone matrix degrading enzyme are observed compared to healthy implants [23]. Therefore, we established cell lines from the human oral cavity, including gingival keratinocytes (GK), osteoblastic lineage cells from the alveolar bone (OLAB), PDL fibroblasts (PDLF), and cementum cells (CC). Using label-free quantitative mass spectrometry, we investigated changes in the proteomes of healthy human oral cells after co-cultivation with *Aggregatibacter actinomycetemcomitans* and *Eikenella corrodens* for 48 h for the first time. Our findings reveal the specific protein profiles for each of the human oral cell lines. This information will improve our understanding of the pathological mechanisms of periodontitis and related diseases, such as rheumatoid arthritis [24], diabetes mellitus [25], and atherosclerotic vascular diseases [26].

## 2. Materials and Methods

Tissue sources: PDL, cementum, alveolar bone, and gingival epithelium were obtained from patients undergoing extraction of premolars or third molars for orthodontic reasons. We only included samples without clinical and radiological signs of periodontitis to ensure that the cultured samples were not contaminated with bacteria before commencing our experimental procedures. Samples from 12 patients were obtained: 6 females and 6 males. Their average age at the time of tooth extraction was 18 years. All patients were healthy nonsmokers and provided written informed consent, consistent with the ethical regulations of our institution (file number: 22/1/05).

Cell isolation and culture: Standard explant cultures of alveolar bone, PDL, cementum, and gingival epithelium samples were performed. We ensured that only the respective tissues were included, without granulomas. All specimens were washed carefully with Braunol (864219, Braun, Melsungen, Germany) three times for 1 min each and then with phosphate-buffered saline (PBS) three times for 1 min each. Afterwards, tissue samples from the PDL or cementum were placed in cell culture dishes with Dulbecco’s Modified Eagle’s Medium (DMEM) + GlutaMAX^TM^ (21885-025, Thermo Fisher, Waltham, WA, USA) supplemented with 10% fetal bovine serum (10270106, Thermo Fisher) and 50 μg/mL gentamycin. After 10 d, outgrown PDLFs and CCs were harvested, and 5 × 10^4^ cells were transferred to 75 cm^2^ flasks (83.1811.002, Sarstedt, Nümbrecht, Germany). Samples of alveolar bone were digested with 0.5 mg of dispase II (17105041, Thermo Fisher) and 1 mg of collagenase (17018029, Thermo Fisher) in 1 mL of DMEM for 12 h. After digestion, the OLABs were released from their matrix using a 40 μm cell strainer (352340, Thermo Fisher). Then, 5 × 10^4^ cells were transferred to a 75 cm^2^ flask. The epithelium of the attached gingiva was separated from the underlying connective tissue by digestion with 2.5 mg of dispase II (17105041, Thermo Fisher) in 1 mL of DMEM for 12 h. Next, epithelial cells were dissociated by incubating the samples with 0.25% trypsin in PBS for 30 min. Afterwards, GKs were seeded on feeder cells that had been inactivated with 50 μg/mL mytomycin C (M0503, Sigma-Aldrich, St. Louis, MO, USA) for 1 h. We used gingival fibroblasts (Han and Amar 2002) obtained from the gingival epithelium samples after the separation of the GKs from the connective tissue as feeder cells. GKs and feeder cells were cultured with Keratinocyte Growth Medium 2 (C-20011, PromoCell, Heidelberg, Germany) supplemented with 0.125 ng/mL EGF, 5 μg/mL insulin, 0.33 μg/mL hydrocortisone, 0.39 μg/mL epinephrine, 10 μg/mL transferrin, 0.004 μL/mL Bovine Pituitary Extract (C-39016, PromoCell), and 0.06 mM CaCl_2_ (C-34006, PromoCell).

Immortalization of oral cell lines, virus production: We seeded 5 × 10^5^ 293T-cells (ACC635, DSMZ, Braunschweig, Germany) into a dish (∅ = 10 cm) and grew them to 80% confluence. On the next day, 10 μg of the hTERT lentiviral plasmid (Bodnar et al. 1998) (customer order, Amsbio, Abingdon, UK) and 10 μg of the packaging plasmid mixture (LV053, ABM, New York, NY, USA) were mixed with 1 mL of DMEM. Furthermore, 80 μL of lentifectin (G074, ABM) were mixed with 1 mL of DMEM. Both solutions were incubated at RT for 5 min, and then they were mixed to allow the transfection complex to form. After 20 min, 4.5 mL of DMEM were added to the transfection complex. The transfection complex was pipetted onto the cells, and 0.65 mL of heat-inactivated FCS was added after 6 h. On the next day, the medium was carefully removed from the cells, and 10 mL of DMEM or Keratinocyte Growth Medium 2 + 10% heat-inactivated FCS + 1% BSA fraction V (BL63-0500, Equitech-Bio, Kerrville, TX, USA) were added. After 24 h, the cells had produced a sufficient amount of the virus, and the supernatant was harvested, centrifuged, and filtered (SLHA033SB, Merck Millipore, Burlington, MA, USA).

Transfection: 1.8 × 10^5^ freshly trypsinized cells were resuspended in 3 mL of virus supernatant and 30 μL of protamine sulfate (P3369, Sigma-Aldrich). Three wells of a 24-well plate were each filled with 1 mL of that solution. After 6 h, 1 mL of medium was added to each well. On the next day, medium and dead cells were removed, and adherent cells received another treatment with 1 mL of the virus supernatant and 10 μL of protamine sulfate per well overnight.

Selection: Infected cells were transferred to a 75 cm^2^ flask and selected by culture with up to 10 μg/mL blasticidin [27].

Culture of bacterial strains: The bacterial strains *Aggregatibacter actinomycetemcomitans* (11122, DSMZ) and *Eikenella corrodens* (8340, DSMZ) were kindly provided by the Department of Microbiology at the Georg August University Goettingen. Bacterial colonies were transferred from an agar plate to an Erlenmeyer flask containing 10 mL of Brain Heart Infusion (BHI) Broth (CM1135, Oxoid, Cheshire, UK) and incubated for 4 days at RT to allow the bacteria to proliferate. Cells were centrifuged and stored in aliquots containing 30% glycerin in BHI to create a stock at −80 °C. For the experiments, one aliquot of each strain was resuspended in BHI at RT.

Cell culture with bacterial strains: We seeded 5 × 10^4^ cells of each human oral cell line at the fourth passage in 75 cm^2^ flasks and grew them to 80% confluence without antibiotics in the respective medium. According to the literature and pilot experiments in our lab, we decided to infect the oral cell lines with 2 × 10^6^ bacterial cells from each strain for 48 h [15,28]. The number of bacteria was determined according to the McFarland standards [29]. After 24 h, human oral cell lines were carefully washed with PBS three times for 1 min each and centrifuged to collect cell pellets.

Cell lysis: Cell pellets were resuspended in a basic lysis buffer consisting of 25 mM Tris, pH 7.4, 0.9% NP-40, 150 mM NaCl, and protease inhibitors (11873580001, Roche, Basel, Switzerland). Afterwards, cells were frozen with liquid nitrogen and thawed five times. The protein concentration was determined with a Pierce BCA Protein Assay Kit (23225, Thermo Fisher, Waltham, WA, USA) and Nanodrop 1000 spectrophotometer (0H517, Thermo Fisher). Protein samples were precipitated with acetone at −20 °C for 12 h.

Immunoblotting: 1.5 × 10^5^ cells were dissolved in 30 μL of 3 × SDS buffer containing 10% β-mercaptoethanol and heated to 95 °C for 5 min. SDS-PAGE was performed with 6% acrylamide in the stacking gel and 8% acrylamide in the resolving gel. After SDS-PAGE, the separated proteins were blotted onto Immobilon-P membranes (PVH07850, Merck Millipore). Total proteins were detected using Coomassie blue staining.

Mass spectrometry sample preparation: Fifty micrograms of protein from each sample were loaded onto a 4–12% NuPAGE Novex Bis-Tris Minigel (NP0329BOX, Thermo Fisher Scientific) and run 1.5 cm into the gel. Following Coomassie staining, the protein bands were excised, diced, and subjected to reduction with dithiothreitol, alkylation with iodoacetamide, and finally overnight digestion with trypsin. Tryptic peptides were extracted from the gel, and the solution was dried in a Speedvac and stored at −20 °C for further analysis [30]. Equal amounts of proteins in aliquots from each sample were pooled to a total of 80 μg and separated into eight fractions using a reverse-phase spin column (84868, Thermo Fisher Scientific) to generate a peptide library. All samples were spiked with a synthetic peptide standard for retention time alignment (Ki-3002-1, Biognosys, Schlieren, Switzerland).

LC/MS/MS analysis: Protein digests were analyzed on a nanoflow chromatography system (Eksigent nanoLC425) connected to a hybrid triple quadrupole-TOF mass spectrometer (TripleTOF 5600+) equipped with a Nanospray III ion source (ion spray voltage 2400 V, interface heater temperature 150 °C, and sheath gas setting 12) and controlled by Analyst TF 1.7.1 software build 1163 (all AB Sciex). Briefly, peptides were dissolved in loading buffer (2% acetonitrile and 0.1% formic acid in water) to a concentration of 0.3 μg/μL. For each analysis, 1.5 μg of digested protein were enriched on a precolumn (0.18 mm ID × 20 mm, Symmetry C18.5 µm; 186000197, Waters, Milford, MA, USA) and separated on an analytical RP-C18 column (0.075 mm ID × 250 mm, HSS T3, 1.8 µm; 186003539, Waters) using a 90 min linear gradient of 5–35% acetonitrile/0.1% formic acid (*v*:*v*) at a rate of 300 nL/min. The qualitative LC/MS/MS analysis was performed using the Top25 data-dependent acquisition method with an MS survey scan of *m*/*z* 350–1250 that accumulated for 350 ms at a resolution of 30,000 full width at half maximum (FWHM). MS/MS scans of *m*/*z* 180–1600 were accumulated for 100 ms at a resolution of 17,500 FWHM and a precursor isolation width of 0.7 FWHM, resulting in a total cycle time of 2.9 s. Precursors exceeding a threshold MS intensity of 125 cps with charge states 2+, 3+, and 4+ were selected for MS/MS, and the dynamic exclusion time was set to 30 s. MS/MS activation was achieved by CID using nitrogen as a collision gas and the manufacturer’s default rolling collision energy settings. Four technical replicates per reverse-phase fraction and a single replicate of each cell co-culture were analyzed to construct a spectral library. For the quantitative SWATH analysis, MS/MS data were acquired using 65 variable size windows across the 400–1050 *m*/*z* range [31]. Fragments were produced using rolling collision energy settings for charge state 2+, and fragments acquired over an *m*/*z* range of 350–1400 for 40 ms per segment. The inclusion of a 100 ms survey scan resulted in an overall cycle time of 2.8 s. Data were acquired from three replicate injections of each biological sample.

Data processing and statistical analysis: Proteins were identified using ProteinPilot Software version 5.0 build 4769 (AB Sciex, Framingham, MA, USA) with “thorough” settings. A total of 689,558 MS/MS spectra from the combined qualitative analyses were searched against the combined UniProtKB *Homo sapiens*, *Aggregatibacter actinomycetemcomitans,* and *Eikenella corrodens* reference proteomes (revision 12-2018, 105,242 entries) augmented with a set of 52 known common laboratory contaminants to identify 2408 proteins with a false discovery rate (FDR) of 1%. Spectral library generation and SWATH peak extraction were performed with PeakView Software version 2.1 build 11041 (AB Sciex) using the SWATH quantitation microApp version 2.0 build 2003. Following retention time correction using the iRT standard, peak areas were extracted using information from the MS/MS library at an FDR of 1% [32], resulting in the quantitation of 2000 proteins across all samples. Protein peak areas were normalized to total area sums (TAS), imported into Perseus v1.5.6.0 software [32,33], and transformed to the log2 scale. A nondirected principal component analysis was performed to examine the reproducibility of biological and technical replicates. Protein peak areas of all ‘Tox’ conditions were compared pairwise to standard conditions using Student’s *t*-tests (*p* < 0.05) and the Benjamini-Hochberg correction for multiple tests. MS raw data, protein identification, and protein quantitation results were deposited in the ProteomeXchange Consortium PRIDE [34] partner repository under the dataset identifier PXD013919. Protein groups that were significantly enriched or depleted were subjected to functional annotation and enrichment analyses using DAVID Bioinformatics Resources 6.8 [35].

## 3. Results

In the present study, we examined the protein expression patterns of GK, OLAB, PDLF, and CC. Each cell-specific proteome was determined in pure culture and after co-cultivation with *Aggregatibacter actinomycetemcomitans* and *Eikenella corrodens* for 48 h. The following cells are marked with a “+” for culture with bacteria or a “−” for culture without bacteria.

### 3.1. Characteristic Phenotypes of Oral Cells

**Figure 1:**
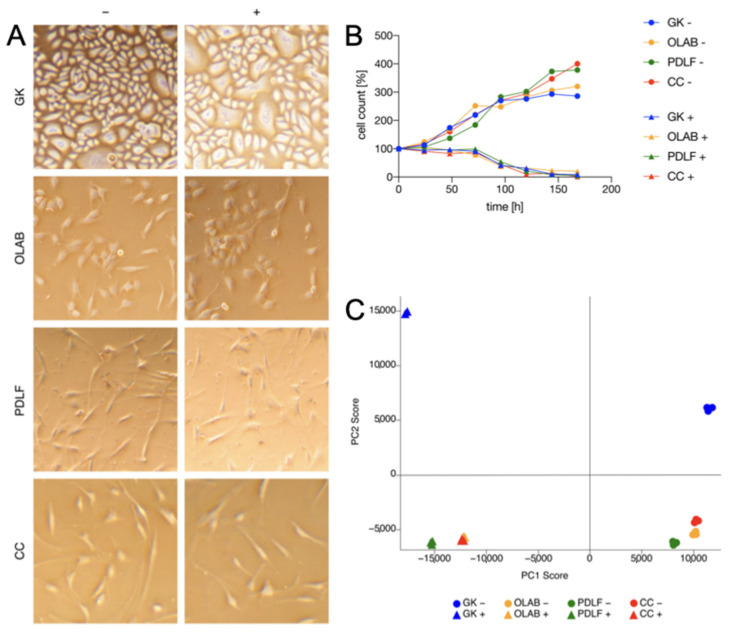
Cell culture and principal component analysis of the investigated oral cells. (**A**) Cytology. Ectodermal GK shows typical epithelial characteristics, such as a cobblestone morphology, high cell density, and direct cell-cell contacts. Mesodermal OLAB, PDLF, and CC exhibit a distinctive fibroblast-like shape. (**B**) Proliferation and apoptosis over 1 week. Culture with bacteria leads to signs of apoptosis after 72 h, (**C**) principal component analysis. Axes reflect the diversity of the proteome. “+” indicates culture with bacteria; “−” indicates culture without bacteria.

GK, an ectodermal cell lineage, showed typical epithelial characteristics, such as a cobblestone morphology, high cell density, and direct cell-cell contacts (Figure 1A, first column). The cells of the mesodermal lineage, including OLAB (Figure 1A, second column), PDLF (Figure 1A, third column), and CC (Figure 1A, fourth column), exhibited a distinctive fibroblast-like shape. OLAB were slightly rounder in shape than PDLF and CC. Compared to OLAB and PDLF, CC showed elongated cell bodies.

Cells that were co-cultivated with *Aggregatibacter actinomycetemcomitans* or *Eikenella corrodens* nor their respective controls showed any signs of apoptosis after 48 h. Morphological differences were not observed between the control specimens (Figure 1A, left panel) and the specimens cultured with bacterial strains (Figure 1A, right panel). The cells cultivated with bacterial strains underwent apoptosis after 72 h (Figure 1B). A principal component analysis (PCA) was performed as a multivariate assessment and revealed distinctive protein patterns for each cell type and for each culture condition (cultivated without or with bacterial strains) (Figure 1C). OLAB, PDLF, and CC represent cells of the mesodermal lineage; generally, they exhibited similar protein expression patterns (Figure 1C, yellow, green, and red dots, respectively, in the lower right area of the graph). Interestingly, their proteomes differed distinctively from the proteome of GK (Figure 1C, blue dots in the upper right area of the graph), which are cells of the ectodermal lineage. Strikingly, all examined cells showed major differences in their proteome patterns after co-cultivation with *Aggregatibacter actinomycetemcomitans* and *Eikenella corrodens* (Figure 1C, yellow, green, red, and blue triangles, respectively, in the left area of the graph). Again, the different lineages of the oral cells were reflected in the different protein patterns (green, red, and blue triangles in the lower left area, blue triangles in the upper left area of the graph, respectively).

### 3.2. Tissue-Specific Characteristics

Below, we will focus on the tissue-specific characteristics of each cell type. The most significant alterations in proteome composition result for the core topics of immune response, cell interactions, and ECM.

#### 3.2.1. GK

**Figure 2:**
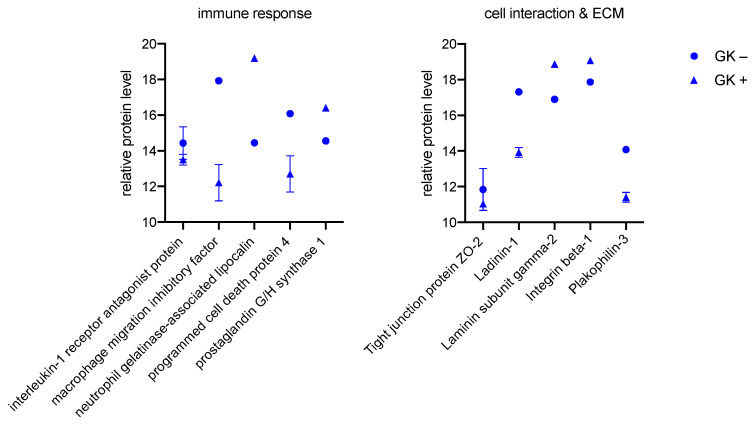
Significant proteins of the immune system (**left**), cell interaction, and ECM (**right**) measured in GK. Protein peak areas were transformed with a log2 scale, and bacterial conditions were compared pairwise with standard conditions using a Student’s *t*-test (*p* < 0.05) and Benjamini-Hochberg correction for multiple tests. “+” indicates culture with bacteria, “−” indicates culture without bacteria.

GK+ exhibit lower protein levels for interleukin-1 receptor antagonist protein, macrophage migration inhibitory factor, programmed cell death protein 4, and increased protein levels for neutrophil gelatinase-associated lipocalin and prostaglandin G/H synthase 1. The adjustments of these proteins, shown on the left in Figure 2, during bacterial culture result in an overall increased immune response.

On the right side of Figure 2, the changes in cell interactions and the ECM are shown. GK+ exhibits reductions in tight junction proteins ZO-2, ladinin-1, and plakophilin-3 compared with GK−, resulting in impaired direct cell interactions. As a component and connection to the basement membrane, laminin subunit gamma-2 are formed more in GK+ and integrin beta-1 is formed les sin GK−.

**Figure 3:**
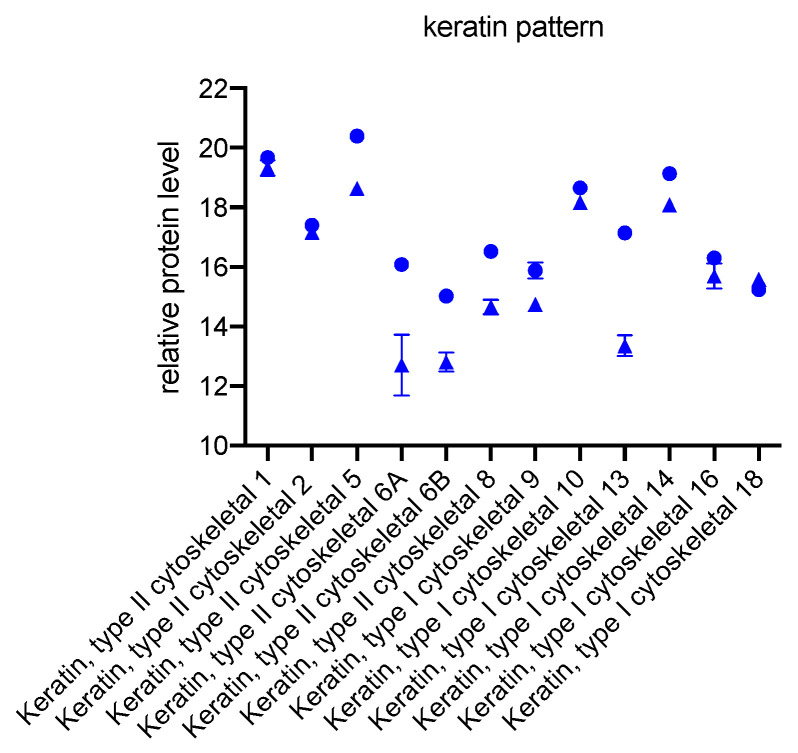
Significant specific expression of keratins in GK. Protein peak areas were transformed with a log2 scale, and bacterial conditions were compared pairwise with standard conditions using a Student’s *t*-test (*p* < 0.05) and Benjamini-Hochberg correction for multiple tests.

The specific expression profile for the keratins in GK is shown separately in Figure 3. GK synthesize keratins 1, 2, 5, 6A, 6B, 8, 9, 10, 13, 14, 16, and 18. During culture with the periodontitis-associated pathogens *Aggregatibacter actinomycetemcomitans* and *Eikenella corrodens*, all previously described keratins are synthesized at significantly reduced levels by GK+ compared with GK−. An exception is keratin 18, which was the only keratin synthesized in increased amounts by GK+ during culture with bacteria.

#### 3.2.2. OBLAs

**Figure 4:**
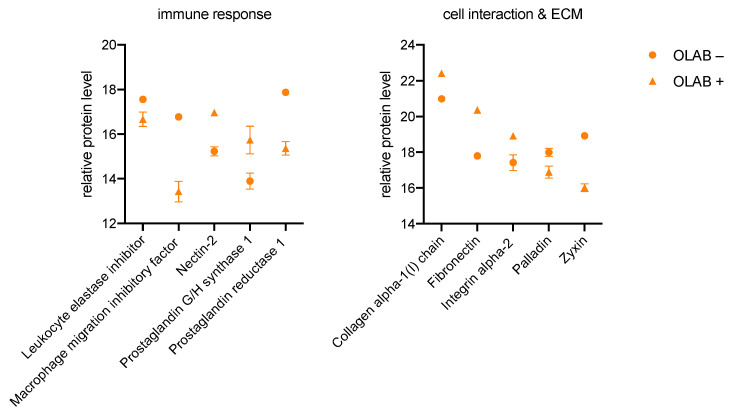
Significant proteins of the immune system (**left**), cell interaction, and ECM (**right**) measured in OLAB. Protein peak areas were transformed with a log2 scale, and bacterial conditions were compared pairwise with standard conditions using a Student’s *t*-test (*p* < 0.05) and Benjamini-Hochberg correction for multiple tests. “+” indicates culture with bacteria; “−” indicates culture without bacteria.

When cultured with bacteria, OLAB+ shows a reduction in leukocyte elastase inhibitor, macrophage migration inhibitory factor, and prostaglandin reductase 1, as well as increased synthesis of nectin-2 and prostaglandin G/H synthase 1, compared with OLAB−. These results in Figure 4, as previously described for GK in Figure 2, result in an increased immune response.

The changes in cell interaction and the ECM, shown on the right in Figure 4, indicate an increase of collagen alpha-1(I) chain, fibronectin, and integrin alpha-2, and a decreased production of palladin and zyxin for OLAB+ compared with OLAB−.

#### 3.2.3. PDLF

**Figure 5:**
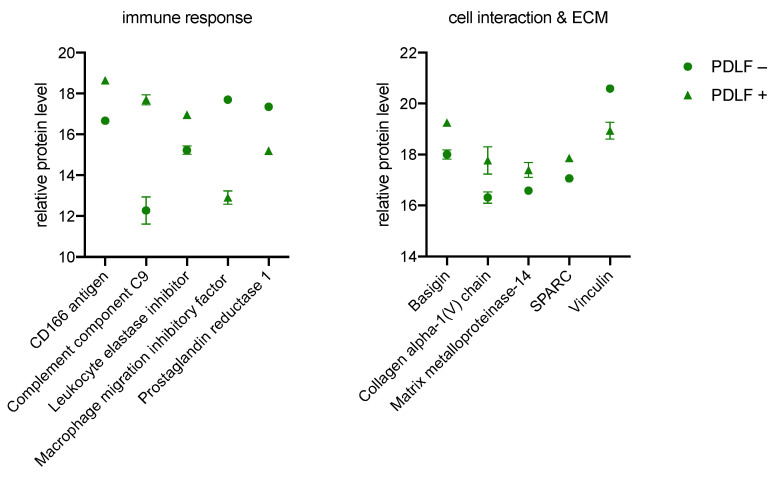
Significant proteins of the immune system (**left**), cell interaction, and ECM (**right**) measured in PDLF. Protein peak areas were transformed with a log2 scale, and bacterial conditions were compared pairwise with standard conditions using a Student’s *t*-test (*p* < 0.05) and Benjamini-Hochberg correction for multiple tests. “+” indicates culture with bacteria; “−” indicates culture without bacteria.

As previously described for GK and OLAB, there are increased and decreased levels of immune-modulating proteins for PDLF, shown in Figure 5. There is increased synthesis of CD166 antigen, complement component C9, and leukocyte elastase inhibitor in PDLF+. Decreased levels of proteins are measured for macrophage migration inhibitory factor and for prostaglandin reductase 1 in PDLF+. Again, an enhancing modulation of the immune response occurs for PDLF+.

At the level of cell interaction and the ECM, there are increased protein levels of basigin, collagen alpha-1(V) chain, matrix metalloproteinase-14, and SPARC in PDLF+, as well as a decreased level of vinculin when compared to PDLF−. Here, bacterial culture causes a restructuring of cell interaction and ECM in PDLF+.

#### 3.2.4. CC

**Figure 6:**
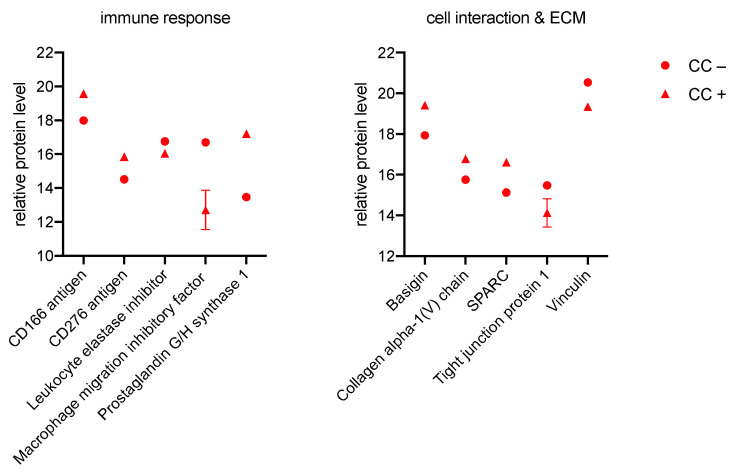
Significant proteins of the immune system (**left**), cell interaction, and ECM (**right**) measured in CC. Protein peak areas were transformed with a log2 scale, and bacterial conditions were compared pairwise with standard conditions using a Student’s *t*-test (*p* < 0.05) and Benjamini-Hochberg correction for multiple tests. “+” indicates culture with bacteria; “−” indicates culture without bacteria.

Even for CC, there is a partial overlap with the previously described cells, shown in Figure 6. The synthesis of CD166 antigen, CD276 antigen, and Leukocyte elastase inhibitor is increased in CC+ compared with CC−. In contrast, a decrease in protein levels is seen for macrophage migration inhibitory factor and prostaglandin G/H synthase 1 in CC+. As with GK, OLAB, and PDLF, this modulation of protein levels results in an enhancement of the immune response.

Alterations of the proteome in the area of cell interaction and ECM are mainly shown by an increased synthesis of basigin, collagen alpha-1(V) chain, and SPARC, as well as a decreased synthesis of tight junction protein 1 and Viculin in CC+. The bacterial pathogens also cause remodeling with a tendency towards degeneration in CC+.

### 3.3. Similar Changes in the Proteome of the Cell Lines during Culture with the Bacteria

**Figure 7:**
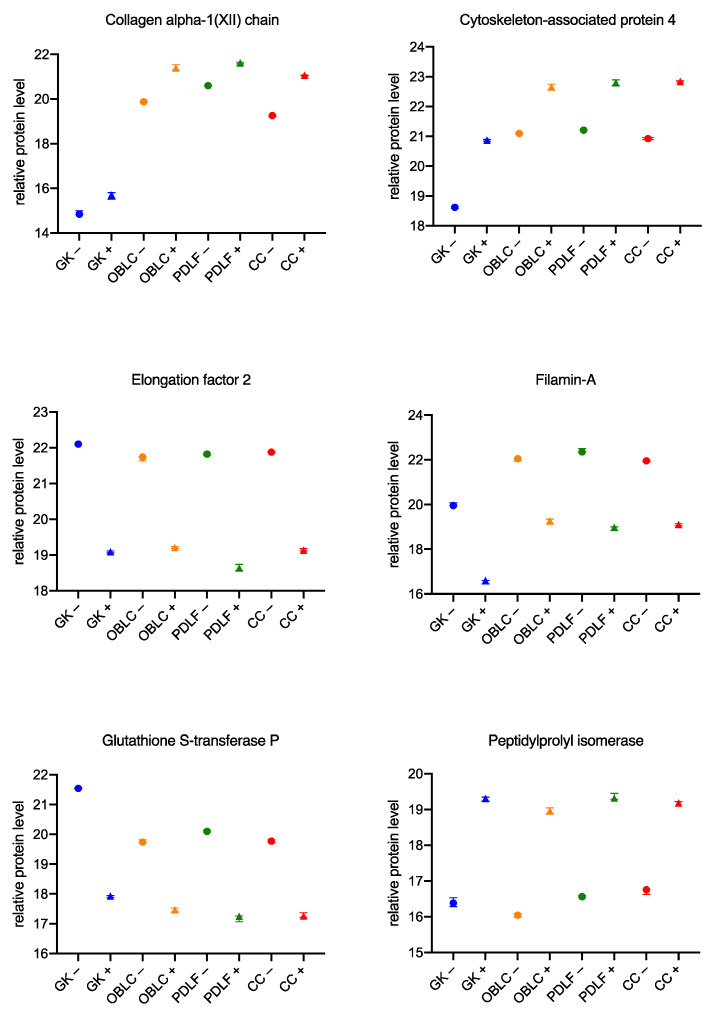
Significant concordances in the behavior of individual cells during culture with bacteria Part 1. Protein peak areas were transformed with a log2 scale, and bacterial conditions were compared pairwise with standard conditions using a Student’s *t*-test (*p* < 0.05) and Benjamini-Hochberg correction for multiple testing. “+” indicates culture with bacteria; “−” indicates culture without bacteria.

**Figure 8:**
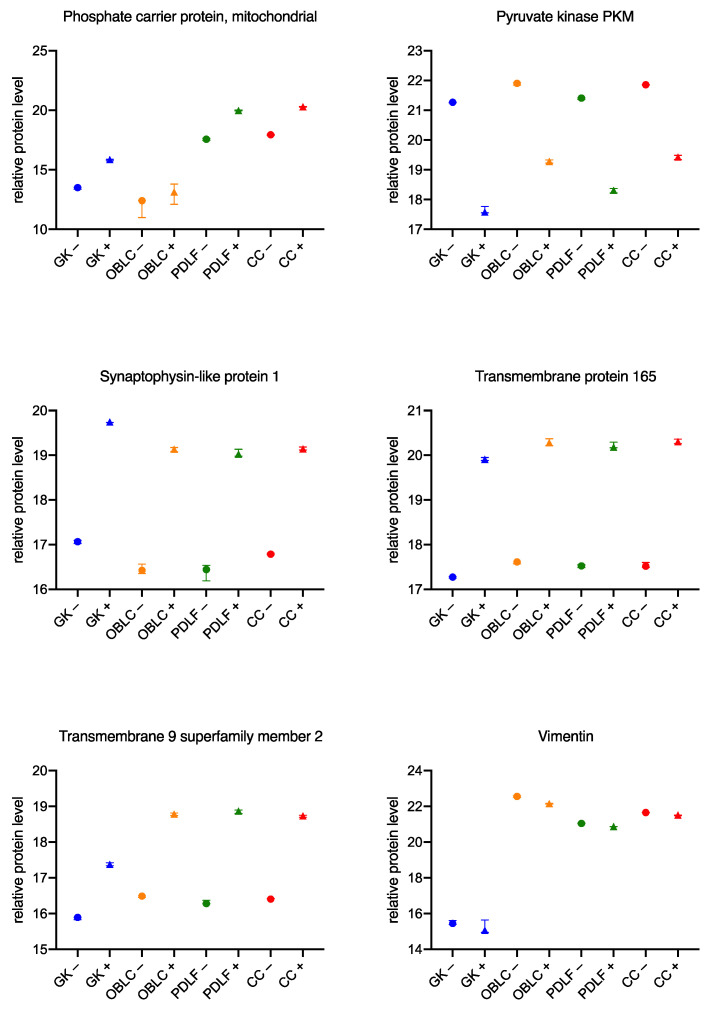
Significant concordances in the behavior of individual cells during culture with bacteria Part 2. Protein peak areas were transformed with a log2 scale, and bacterial conditions were compared pairwise with standard conditions using a Student’s *t*-test (*p* < 0.05) and Benjamini-Hochberg correction for multiple testing. “+” indicates culture with bacteria; “−” indicates culture without bacteria.

Previously, mainly differences in the individual cell series during culture with *Aggregatibacter actinomycetemcomitans* and *Eikenella corrodens* were considered. In order to generate possible therapeutic approaches, the similarities between the different cells should also be considered so that a potential therapeutic candidate can be identified and further investigated. Figure 7 and Figure 8, divided for a better overview, demonstrate the 12 most differentially expressed proteins.

In the upper left of Figure 7, an increase of the collagen alpha-1 (XII) chain in GK+, OLAB+, PDLF+, and CC+ can be seen compared to the corresponding cell series in culture without bacteria. A similar increase in protein level is observed for cytoskeleton-associated protein 4 (top right of Figure 7) and for Peptidylprolyl isomerase (bottom left of Figure 7) in the cells cultured with bacteria. A reduction of protein levels in GK+, OLAB+, PDLF+, and CC+ compared with GK−, OLAB−, PDLF−, and CC− could be measured for elongation factor 2 (center left in Figure 7), filamin-A (center right in Figure 7), and glutathione S-transferase P (bottom left in Figure 7).

During culture with the PA-associated bacteria *Aggregatibacter actinomycetemcomitans* and *Eikenella corrodens*, there is an increase in relative protein levels in GK+, OLAB+, PDLF+, and CC+ for phosphate carrier protein mitochondrial (top left of Figure 8), synaptophysin-like protein 1 (center left of Figure 8), transmembrane protein 165 (center right of Figure 8), and transmembrane 9 superfamily member 2 (bottom left of Figure 8). In contrast, for pyruvate kinase (PKM; top right of Figure 8) and vimentin (bottom right of Figure 8), decreased relative protein levels are measured in GK+, OLAB+, PDLF+, and CC+ compared with GK−, OLAB−, PDLF−, and CC−.

## 4. Discussion

In the present study, we investigated the protein expression patterns of GK, OLAB, PDLF, and CC using state-of-the-art label-free mass spectrometry. For the first time, we describe cell-specific protein expression patterns and specific changes in these patterns upon co-cultivation with *Aggregatibacter actinomycetemcomitans* and *Eikenella corrodens*.

### 4.1. The Proteome of Mesodermal Lineage Cells Vary Distinctly from Those of Ectodermal Lineage

OLAB, PDLF, and CC show specific but similar protein patterns in PCA. Comparable results were shown in mesodermal cells and their derivatives [36]. Remarkably, analogous protein patterns of the investigated cells from the mesodermal lineage were shown after bacterial irritation [37]. These results suggest that bacterial irritation of different mesodermal cells leads to similar metabolic responses.

As expected, large differences were observed in the protein pattern of GK from the ectodermal lineage compared with the group consisting of OLAB, PDLF, and CC of mesodermal origin. The protein pattern of GK is significantly altered after bacterial irritation, but with remaining differences from cells of the mesodermal lineage. In a multi-omics profiling experiment, similar findings regarding the differences between ectodermal and mesodermal cells could be described [38].

### 4.2. Changes in Keratin Pattern

The performed proteome analysis showed that GK are completely different from OLAB, PDLF, and CC. Although oral keratinocyte lines are scarce, it is known that they express keratins 5, 6, 7, 8, 13, 14, 16, 17, 18, and 19 in cell culture [10]. In addition to those, keratins 1, 2, and 10 were expressed by GK in the present study. Interestingly, we observed a decrease in keratin levels in GK after cultivation with the two bacterial strains, while an increase of keratins 1, 10, and 14 after cultivation with *Porphyromonas gingivalis* has been described [39]. Proteomic analyses of gingival crevicular fluid are consistent with our findings. A pattern of keratins, including keratins 1, 2, 3, 9, and 10 [40], was found. A decrease in keratin levels during bacterial irritation is clearly seen in the present investigation, consistent with an impaired mechanical barrier. Keratin 18 was the only keratin to increase during culture with bacteria. Such an increase of keratin 18 has been shown previously in colorectal carcinomas [41].

### 4.3. Modulation of an Enhanced Immune Response in All Investigated Cells

Signs of increased immune activation in GK were found in upregulated protein levels of neutrophil gelatinase-associated lipocalin [42], programmed cell death protein 4 [43], and downregulated levels of interleukin-1 receptor antagonist protein [44]. Macrophage migration inhibitory factor was downregulated in all cells examined, indicating increased macrophage activity. Increased levels of macrophage migration inhibitory factor were described in chronic periodontitis [45]. Prostaglandin G/H synthase 1, which is known to produce the inflammatory prostaglandin E2 [46], was increased in GK, CC, and OLAB. Decreases in prostaglandin reductase 1 in OLAB and PDLF after bacterial irritation lead to higher leukotriene B4 levels and thus increase the inflammatory response [47]. Down-regulation of leukocyte elastase inhibitor in mesodermal cells is also consistent with an activated immune response, in which more elastase is released from leukocytes [48]. Binding of CD166 antigen to CD6 promotes activation of the acquired immune system by T cells [49]. CD166 antigen expression was increased in PDLF and CC after bacterial irritation. Similarly, nectin-2, a modulator of T cell signaling [50], was increased in OLAB. In CC, the CD276 antigen, also associated with T-cell activation and IFN amplification [51], was increased. Complement component C9 was elevated in PDLF, indicating an enhanced innate immune response [52].

### 4.4. Proteins of Cell Interaction and ECM Are Affected by Culture with Bacteria

The reductions of tight junction protein ZO-2 [53], ladinin-1 [54], and plakophilin-3 [55] in GK, of paladin [56] and zyxin [57] in OALB, of tight junction protein 1 [53] in CC, and of vinculin [57] in PDLF and CC suggest impaired cell interaction and reduced ECM integrity. Similarly, increased protein levels of matrix metalloproteinase-14 and its activator basigin, typical of inflammation, in PDLF reflect the degradation of ECM [58]. In this light, the increases of laminin subunit gamma-2 [59] and integrin beta-1 [60] in GK, of collagen alpha-1(I) chain [1], fibronectin [61], and integrin alpha-2 [60] in OLAB, and of collagen alpha-1(V) chain [1] in PDLF and CC appear contradictory. Presumably, degradation and regeneration processes occur simultaneously during simulated inflammation in vitro [62].

### 4.5. Similar Changes in the Proteome across Cell Lines

Common changes in the proteomes of GK, OLAB, PDLF, and CC are of therapeutic importance to identify potential candidates, which would then need to be intensified for further investigation [63].

Collagen alpha-1 (XII) is responsible for the structure of the ECM. It is produced in high levels by all cells during bacterial culture. The PDL of a mouse with mutations in collagen alpha-1 (XII) demonstrated a loss of the ordered architecture of the PDL, without evidence of periodontitis [64]. Therefore, modulation of collagen alpha-1 (XII) does not seem to be targetable in terms of a new therapeutic approach for periodontitis. Cytoskeleton-associated protein 4 regulates the exocytosis of proteases, lipases, and inflammatory mediators from neutrophil granulocytes [65]. It is increasingly synthesized during bacterial culture and has been implicated in the immune response. Increased expression, as in the experiment, seems to be useful as part of the immune system, but on the other hand, it contributes to periodontal degradation. Elongation factor 2 catalyzes the coordinated movement of tRNA molecules. In conclusion, as in the experiment shown, protein biosynthesis is reduced by the lack of elongation factor 2 [66]. This seems to represent a possible intervention for a local therapy for periodontitis. Filamin-A is decreased during bacterial culture by GK, OLAB, PDLF, and CC. The roles of filamin-A are diverse and involve various interaction proteins. Most importantly, the role in neoplasia development, which is not precisely elucidated [67], leaves a therapeutic benefit for periodontitis therapy in the background. Glutathione S-transferase P serves to regenerate the cell [68]. A study with 60 subjects describes mutations of glutathione S-transferase P as a risk factor for chronic periodontitis [69]. FKBP11 is elevated in cells subjected to bacterial irritation. As an isomerase, it probably has little relevance to periodontitis therapy, although elevated levels have already been detected in patients with chronic periodontitis [70]. The phosphate carrier protein mitochondrial is ubiquitous, conserved through evolution, and overall, a non-influencing factor in this experiment or during periodontitis. Deficiencies in the phosphate carrier protein mitochondria are lethal within the first year of life [71]. Pyruvate kinase PKM is produced at a reduced level during bacterial culture in the experiment described. Potential therapeutic approaches for periodontitis need to be carefully evaluated, as there is an unclear association with oral squamous cell carcinoma [72]. Increased synptophysin-like protein 1 is involved in the release of neurotransmitters from the synapse [73]. Therapeutic relevance does not exist in the current low data situation. Increased levels of Transmembrane 9 superfamily member 2 were detected in all cells with bacterial cultures. An association with periodontitis exists at the genetic level [74]. It is presented as a potential oncogene and is expected to become a potential drug target for newer drugs [75]. It is quite conceivable that results from research on Transmembrane 9 superfamily member 2 as a new oncogene can also be applied to the therapy of periodontitis. Vimentin, as an intermediate filament within the cell, is reduced in production due to bacterial irritation. During periodontitis, most collagenous structures are degraded. Vimentin appears to have a protective effect on the stability of collagen mRNAs [15,76]. Thus, it could also represent a potential target site for therapies to maintain the biosynthesis of collagen. However, vimetin should be investigated more deeply as it plays a role in the epithelial–mesenchymal transition [77].

## 5. Conclusions

Periodontitis-like conditions were simulated by the co-cultivation of cells with two microorganisms, while periodontitis is triggered by a complex biofilm involving several bacteria and an immune response of the respective infected host organism in vivo. Therefore, our experimental procedures do not claim to be an accurate simulation of in vivo periodontitis conditions. Nevertheless, *Aggregatibacter actinomycetemcomitans* and *Eikenella corrodens* drastically alter the protein expression patterns of infected cells, even outside of an organized biofilm. Further investigations with multispecies biofilms will be required to translate our findings to in vivo conditions. The present study will help improve our understanding of the pathological mechanisms of periodontitis and may aid in the elucidation of new treatment options that focus on influencing cellular mechanisms. Remarkably, we established a pool of human oral cell lines from attached gingiva, alveolar bone, PDL, and cementum that will be available for future in vitro periodontitis research.

## Data Availability

MS raw data, protein identification, and protein quantitation results were deposited in the ProteomeXchange Consortium PRIDE [34] partner repository under dataset identifier PXD013919.
